# Right Ventricular Longitudinal Shortening is not Associated with Left Ventricular Rotational Mechanics in Healthy Adults - Insights from the Three-dimensional Speckle-tracking Echocardiographic MAGYAR-Healthy Study

**DOI:** 10.31083/j.rcm2502053

**Published:** 2024-02-02

**Authors:** Attila Nemes, Árpád Kormányos, Zoltán Ruzsa, Alexandru Achim, Nóra Ambrus, Csaba Lengyel

**Affiliations:** ^1^Department of Medicine, Albert Szent-Györgyi Medical School, University of Szeged, H-6725 Szeged, Hungary

**Keywords:** left ventricular, rotation, tricuspid annular plane systolic excursion, right atrial, volume, three-dimensional, echocardiography

## Abstract

**Background:**

The left ventricle (LV) not only contracts, but its rotational mechanics have a significant role in systolic ejection, whereas the right ventricle (RV) is substantially different in shape and function, and its contractility is not accompanied by rotational features. Simple M-mode echocardiography-based tricuspid annular plane systolic excursion (TAPSE) reflects RV longitudinal contraction or shortening. The aim of the present study was to examine the relationship between the parameters characterizing the rotational mechanics of the LV as assessed by three-dimensional speckle-tracking echocardiography (3DSTE) and the TAPSE. The effects of different degrees of these parameters on each other were also examined.

**Methods:**

The present retrospective analysis evaluated the results of 80 healthy adult individuals with an average age of 28.1 ± 6.3 years (33 males) with LV rotational mechanics being directed normally. All cases have undergone complete two-dimensional Doppler echocardiography with the measurement of TAPSE and 3DSTE.

**Results:**

None of the LV volumes and rotational parameters showed any differences in healthy cases with TAPSE 18–21 mm vs. TAPSE >22 mm. Similarly, right atrial (RA) volumetric parameters did not differ either. TAPSE showed no associations with the degree of basal LV rotation. RA volumes were slightly increased with higher basal LV rotation. Similar to basal LV rotation, TAPSE did not change with the degree of apical LV rotation and a tendentious increase of RA volumes could be demonstrated with increasing apical LV rotation. No correlation could be demonstrated between apical and basal LV rotations and TAPSE.

**Conclusions:**

3DSTE-derived LV rotational parameters and TAPSE are not associated suggesting that LV twist is independent of RV longitudinal shortening in healthy circumstances.

## 1. Introduction

Modern echocardiographic techniques help to perform a detailed analysis of the cardiac mechanics of both ventricles allowing physiological assessments. Although the two types of movement cannot be separated, the walls of the left ventricle (LV) not only contract during the cardiac cycle represented by deformation parameters, but the walls have their rotational mechanics as well [1, 2]. It has been demonstrated that LV rotational mechanics contribute significantly to LV ejection [1, 3]. The right ventricle (RV) is substantially different in shape and function compared to the LV, but its contractility is not accompanied by rotational features [4, 5]. The long-used and validated, simple M-mode echocardiography-derived tricuspid annular plane systolic excursion (TAPSE) reflects RV longitudinal contraction or shortening [6, 7, 8, 9, 10]. Whereas LV and RV functions differ, comparing their subfunctions could help us understanding the physiology of their interdependence and interplay [11]. The aim of this study was to examine the relationship between the parameters characterizing the rotational mechanics of the LV as assessed by three-dimensional speckle-tracking echocardiography (3DSTE) and the TAPSE, a quantitative feature of RV longitudinal shortening in real clinical settings by imaging methods in healthy subjects. We examined the effects of different degrees of these parameters on each other as well.

## 2. Subjects and Methods

### 2.1 Subjects

The present retrospective analysis comprised 80 healthy adult volunteers with a mean age of 28.1 ± 6.3 years (33 males) with normally directed LV rotational mechanics. All participants underwent physical examination, laboratory testing, standard 12-lead electrocardiography (ECG) and two-dimensional (2D) Doppler echocardiography with a negative result. None of the participants were taking any medication, none were smokers or obese, and none had a known disorder or any pathological state. 3DSTE-derived data acquisition was performed directly after the 2D echocardiographic examination in accordance with recent practices and detailed 3DSTE-derived analysis was performed at a later date offline. The present retrospective cohort study is part of the **‘M**otion **A**nalysis of the heart and **G**reat vessels b**Y **three-dimension**A**l speckle-t**R**acking echocardiography in **Healthy **subjects’** (MAGYAR-Healthy**)** Study**. This study was organized to examine the physiologic relationship between different volumetric and functional parameters including 3DSTE-derived variables in healthy circumstances (‘Magyar’ means ‘Hungarian’ in Hungarian language). The study was approved by the Institutional and Regional Human Biomedical Research Committee of University of Szeged, Hungary (No.: 71/2011) and all healthy volunteers gave informed consent. The study was conducted in accordance with the Declaration of Helsinki (as revised in 2013).

### 2.2 Two-Dimensional Doppler Echocardiography

**Fig. 1.**
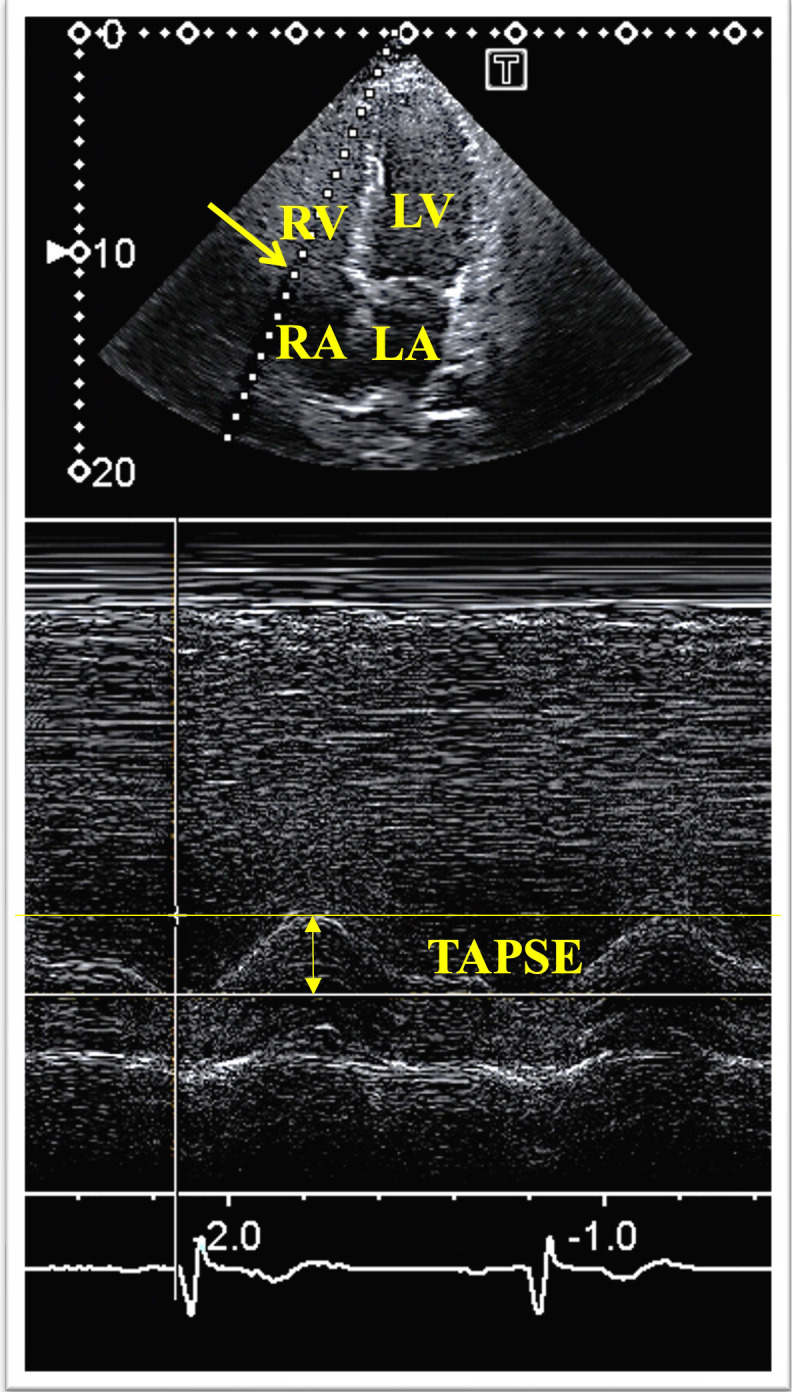
**M-mode echocardiography-derived measurement of tricuspid annular plane systolic excursion (TAPSE)**. Abbreviations: TAPSE, tricuspid annular plane systolic excursion; RA, right atrium; RV, right ventricle; LA, left atrium; LV, left ventricle.

Professional guidelines and accepted practices were followed for 2D Doppler cardiac ultrasound measurements. A Toshiba ${\mathrm{Artida}}^{\text{TM}}$ cardiac ultrasound tool (Toshiba Medical Systems, Tokyo, Japan) attached to a 1–5 MHz PST-30BT phased-array transducer was used for all measurements. Subjects were lying on their left side, and then, the transducer was placed on the chest and measurements were made from the typical parasternal and apical views. Doppler echocardiography was used to exclude any significant degree of valvular stenosis and regurgitation on any valves, and transmitral E and transmitral A flow velocities and E/A were determined to evaluate the diastolic function of the LV. Chamber quantifications including left atrial and LV measurements were also performed with the measurement of the LV ejection fraction (EF) according to the Simpson’s method [12]. TAPSE, systolic longitudinal TA motion as a representation of RV longitudinal shortening was assessed in apical long-axis view as a movement of the lateral edge of the TA toward the apex in systole [6, 7, 8, 9, 10] (Fig. 1).

### 2.3 Three-Dimensional Speckle-Tracking Echocardiography

During 3DSTE studies, the same Toshiba ${\mathrm{Artida}}^{\text{TM}}$ cardiac ultrasound tool (Toshiba Medical Systems, Tokyo, Japan) was used by the same observer (ÁK), and a PST-25SX matrix-array transducer was attached to the ultrasound tool. For 3DSTE, all subjects had to be in sinus rhythm and the subjects were lying in the left lateral position. Six 3D echocardiographic subvolumes (datasets) were then acquired within 6 cardiac cycles with respiratory retention from the apical window, the subvolumes were merged together automatically for a full-volume dataset. At a later date, data analysis was performed with a 3D Wall Motion Tracking software version 2.7 (Ultra Extend, Toshiba Medical Systems, Tokyo, Japan) [13, 14, 15, 16].

### 2.4 3DSTE-Derived LV Rotational Mechanics

**Fig. 2.**
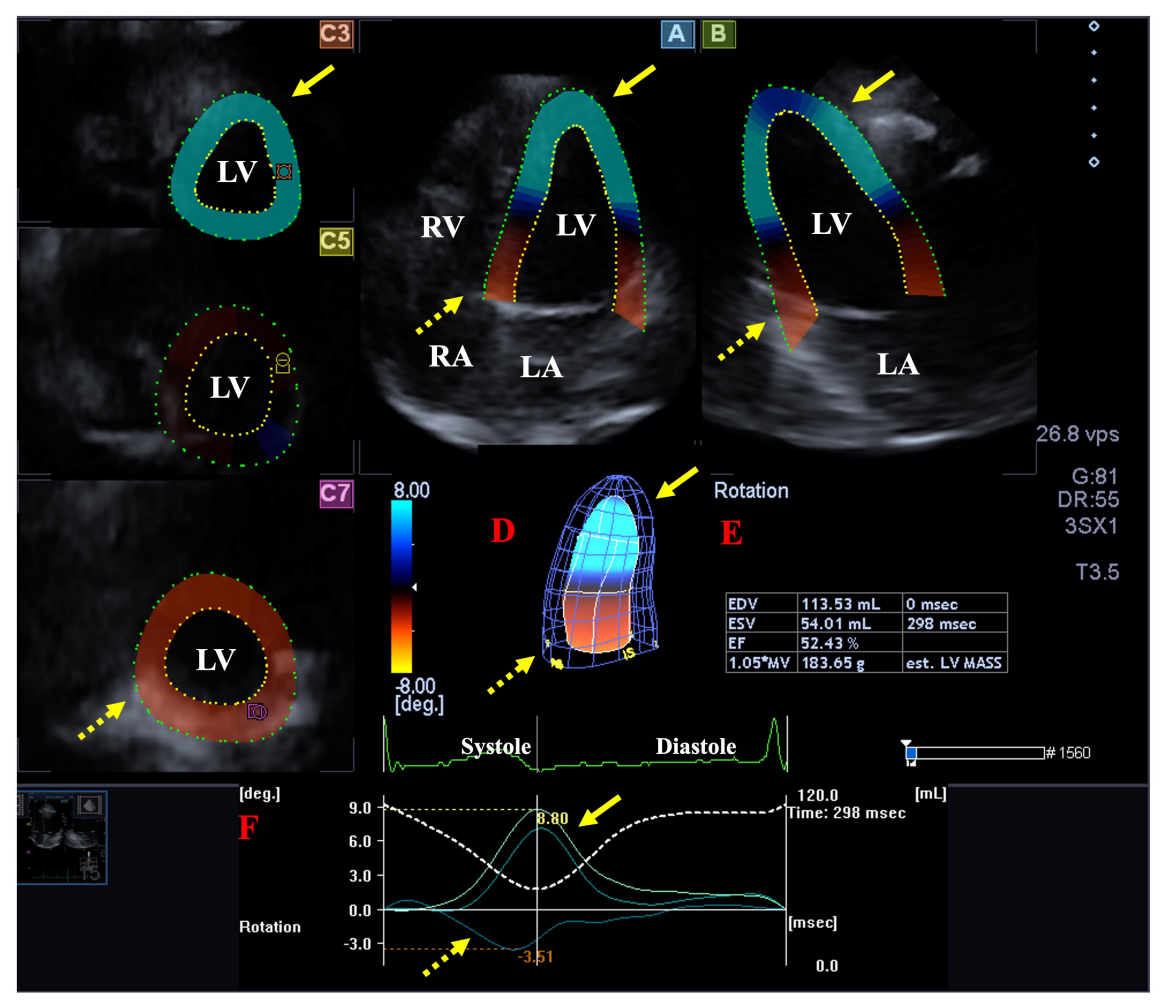
**Analysis of the rotational mechanics of the left ventricle (LV) by three-dimensional (3D) speckle-tracking echocardiography**. Apical longitudinal four-chamber (A) and two-chamber views (B) and short-axis views at apical (C3), midventricular (C5) and basal LV levels (C7) are shown together with a 3D model of the LV (D) and calculated LV volumetric data (E). Time - LV apical (yellow arrows) and basal (dashed yellow arrows) rotation curves are shown together with time - LV volume change curve during the cardiac cycle (F). Abbreviations: EDV, end-diastolic volume; ESV, end-systolic volume; EF, ejection fraction; RA, right atrium; RV, right ventricle; LA, left atrium; LV, left ventricle.

Apical longitudinal 4-chamber (AP4CH) and 2-chamber (AP2CH) views and apical, midventricular and basal cross-sectional planes were automatically selected by the software during analysis. Mitral annular (MA)-LV lateral and septal edges and the endocardial surface of the LV apex were defined. Then a sequential analysis was started to create a virtual 3D cast of the LV helping to determine the following LV rotational parameters using a 3D cast of the LV [17] (Fig. 2):

- clockwise basal LV rotation (in degrees).

- counterclockwise apical LV rotation (in degrees).

- LV twist (net difference of LV apical and basal rotations in degrees).

- time-to-peak LV twist (in milliseconds).

There is a special form of LV rotational mechanics when the direction of LV apical and basal rotations are in the same clockwise/counterclockwise direction, therefore LV twist cannot be measured, and only an LV apico-basal gradient is present (so-called LV ‘rigid body rotation’, RBR). Subjects with LV-RBR were not involved in this study [18].

### 2.5 3DSTE-Derived Assessment of RA Volumes

**Fig. 3.**
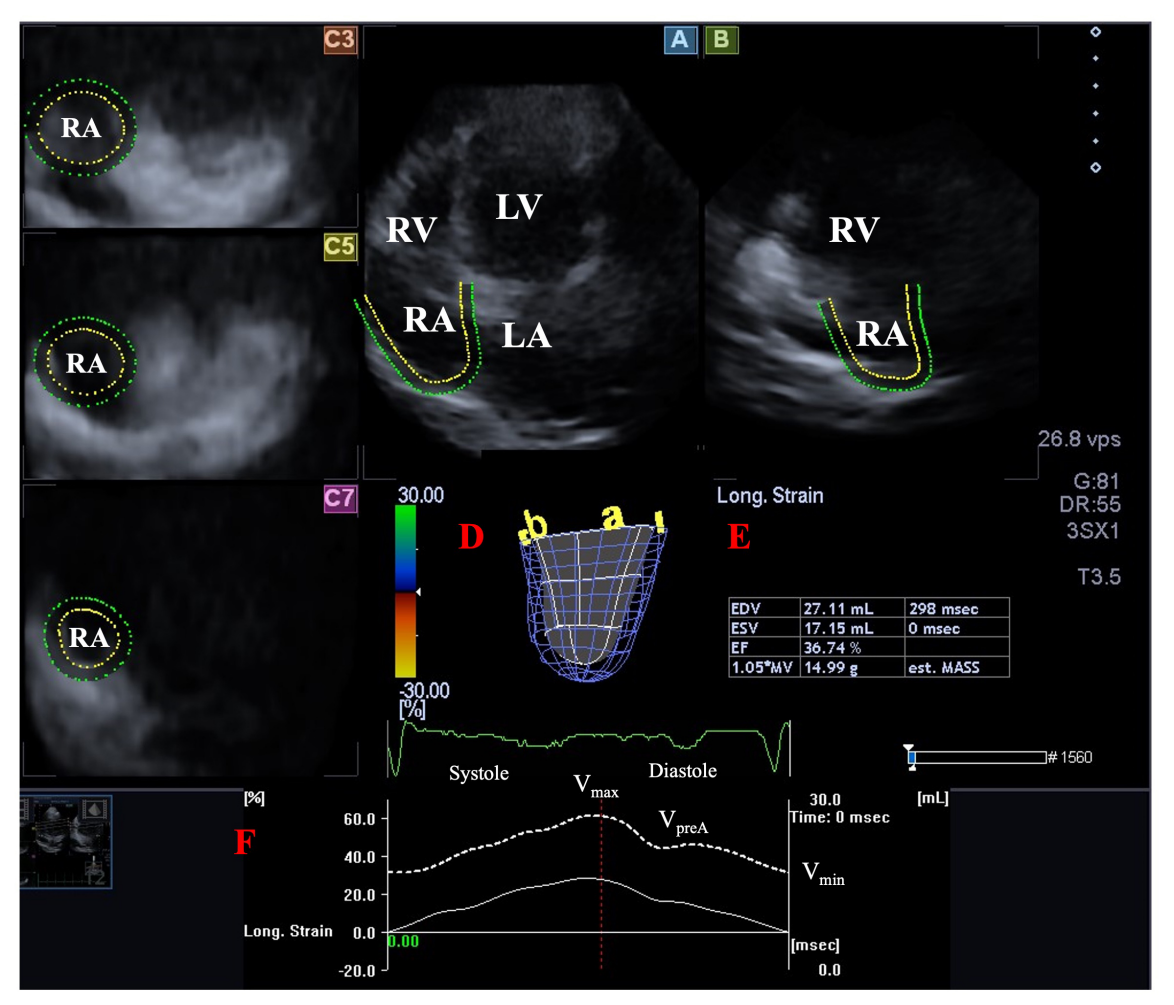
**Assessment of the right atrium by three-dimensional (3D) speckle-tracking echocardiography**. Apical four-chamber (A) and two-chamber views (B) and short-axis views at basal (C3), midatrial (C5) and superior RA levels (C7) are demonstrated together with a 3D RA cast (D) and calculated RA volumes (E). Time - RA volume changes curve represented by a white dashed line is also shown (F). Abbreviations: LA, left atrium; LV, left ventricle; RA, right atrium; RV, right ventricle; EDV, end-diastolic volume; ESV, end-systolic volume; EF, ejection fraction; $V_{\text{max}}$, maximum RA volume; $V_{\text{preA}}$, pre-atrial contraction RA volume; $V_{\text{min}}$, minimum RA volume.

In RA-focused images, similarly to the images focusing on the LV, data were displayed in selected apical longitudinal two- (AP2CH) and four-chamber (AP4CH) views and 3 short-axis views at basal, midatrial and superior levels at end-diastole. A 3D model of the RA was created following the definition of septal and lateral RA-TA edges and RA apex in AP2CH and AP4CH views at end-diastole, then the endocardial RA surface was reconstructed with sequential analysis. The following RA volumes were obtained [19] (Fig. 3):

- maximum RA volume, measured at end-systole, just before the opening of the tricuspid valve ($V_{\text{max}}$).

- RA volume before atrial contraction, measured at early-diastole at the time of the P wave on the ECG ($V_{\text{preA}}$).

- minimum RA volume measured at end-diastole, just before the closure of the mitral valve ($V_{\text{min}}$).

### 2.6 Statistical Analysis

Mean ± standard deviation (SD) (for continuous variables) and *n* (%) format (for categorical variables) were used for data presentations. Statistical significance was considered to be present when *p* < 0.05. Pearson’s coefficients were measured to characterize correlations between variables. Analysis of variance (ANOVA) and independent sample *t*-test were used for the comparison of groups. SPSS software analyses (SPSS Inc. version 22, Chicago, IL, USA) were performed.

## 3. Results

### 3.1 Clinical and Two-Dimensional Doppler Echocardiographic Data

Normal clinical and routine 2D Doppler echocardiographic parameters were found in all cases as presented in Table 1.

**Table 1.** **Clinical and two-dimensional echocardiographic data**.

Data		Measurements
Clinical data		
	n	80
	Mean age (years)	28.1 ± 6.3
	Males (%)	33 (41%)
	Systolic blood pressure (mmHg)	120.8 ± 4.0
	Diastolic blood pressure (mmHg)	78.4 ± 3.1
	Heart rate (1/s)	71.2 ± 1.9
	Height (cm)	170.2 ± 10.1
	Weight (kg)	72.8 ± 15.3
	Body surface area (${\mathrm{kg/m}}^{2}$)	1.86 ± 0.33
Two-dimensional echocardiographic data		
	LA diameter (mm)	36.9 ± 3.2
	LV end-diastolic diameter (mm)	48.1 ± 3.6
	LV end-systolic diameter (mm)	32.4 ± 3.5
	LV end-diastolic volume (mL)	105.8 ± 23.8
	LV end-systolic volume (mL)	38.3 ± 9.5
	Interventricular septum (mm)	9.1 ± 1.2
	LV posterior wall (mm)	9.2 ± 1.4
	LV ejection fraction (%)	64.5 ± 4.2
	Early diastolic mitral inflow velocity - E (cm/s)	82.8 ± 15.0
	Late diastolic mitral inflow velocity - A (cm/s)	55.6 ± 10.7

Abbreviations: LA, left atrial; LV, left ventricular.

### 3.2 Classification of Subjects

According to guidelines, TAPSE is considered to be normal if ≥17 mm, while the average TAPSE proved to be approximately 21.5 mm in healthy individuals in a recent study [8, 10]. Therefore, the group of healthy subjects was divided into 2 subgroups: subjects with TAPSE between 17 and 21 mm were compared with subjects with TAPSE ≥22 mm. For another comparison, subjects were divided into subgroups according to the mean ± SD of basal and apical LV rotations as assessed by 3DSTE as well, the values were –4.10 ± 2.31° and 9.37 ± 4.04°, respectively. The healthy subjects were classified into subgroups based on the lower (–1.79° and 5.33°, respectively) and upper (–6.41° and 13.41°, respectively) values of these parameters.

### 3.3 Degree of TAPSE and LV Rotational Mechanics

No difference was found in the case of LV volumes and the rotational parameters in healthy cases with TAPSE 18–21 mm vs. TAPSE >22 mm. Similarly, RA volumetric parameters did not differ either (Table 2).

**Table 2.** **Tricuspid annular plane systolic excursion, volumetric and rotational parameters of the left ventricle and right atrial volumes in different tricuspid annular plane systolic excursion groups**.

	All subjects	TAPSE 18–21 mm	TAPSE ≥22 mm
	(n = 80)	(n = 21)	(n = 59)
LV-EDV (mL)	85.7 ± 20.5	82.4 ± 23.2	86.9 ± 19.4
LV-ESV (mL)	36.2 ± 10.1	34.4 ± 11.9	36.8 ± 9.3
LV-EF (%)	57.9 ± 5.7	58.9 ± 6.4	57.6 ± 5.4
LV mass (g)	164.2 ± 31.4	161.3 ± 27.6	165.2 ± 32.6
LV basal rotation (°)	–4.10 ± 2.31	–4.20 ± 2.16	–4.06 ± 2.35
LV apical rotation (°)	9.37 ± 4.04	9.40 ± 3.41	9.36 ± 5.25
LV twist (°)	13.47 ± 4.43	13.60 ± 3.71	13.42 ± 4.66
time-to-LV twist (ms)	354 ± 128	376 ± 130	346 ± 126
${\mathrm{RA-V}}_{\text{max}}$ (mL)	50.4 ± 14.8	47.0 ± 15.2	51.5 ± 14.5
${\mathrm{RA-V}}_{\text{preA}}$ (mL)	35.2 ± 10.0	33.4 ± 9.9	35.8 ± 10.0
${\mathrm{RA-V}}_{\text{min}}$ (mL)	27.4 ± 9.3	25.8 ± 9.9	27.9 ± 9.1
TAPSE (mm)	23.7 ± 3.0	20.3 ± 0.8	24.9 ± 2.5*

*p < 0.05 vs. TAPSE 18–21 mm; Abbreviations: LV, left ventricular; EDV, end-diastolic volume; ESV, end-systolic volume; EF, ejection fraction; RA, right atrial; $V_{\text{max}}$, end-systolic maximum RA volume; $V_{\text{preA}}$, early diastolic preatrial contraction RA volume; $V_{\text{min}}$, end-diastolic minimum RA volume; TAPSE, tricuspid annular plane systolic excursion.

### 3.4 Degree of Basal LV Rotation and TAPSE

TAPSE showed no association with the degree of basal LV rotation. A tendentious increase of LV-ESV and a decrease of LV-EF could be detected with higher basal LV rotation. Moreover, RA volumes showed a slight increase as well (Table 3).

**Table 3.** **Tricuspid annular plane systolic excursion and rotational parameters of the left ventricle in different left ventricular basal and apical rotation groups**.

	basal ${\mathrm{LV}}_{\text{rot}}$ < –1.79°	–1.79° ≤ basal ${\mathrm{LV}}_{\text{rot}}$ ≤ –6.41°	–6.41° < basal ${\mathrm{LV}}_{\text{rot}}$	apical ${\mathrm{LV}}_{\text{rot}}$ < 5.33°	5.33° ≤ apical ${\mathrm{LV}}_{\text{rot}}$ ≤ 13.41°	13.41° < apical ${\mathrm{LV}}_{\text{rot}}$
	(n = 9)	(n = 58)	(n = 13)	(n = 12)	(n = 54)	(n = 14)
LV-EDV (mL)	87.8 ± 14.7	85.6 ± 22.2	88.2 ± 15.3	83.2 ± 13.7	86.8 ± 21.8	83.6 ± 20.0
LV-ESV (mL)	32.9 ± 6.2	35.7 ± 10.6	40.8 ± 8.0*	36.7 ± 6.6	36.8 ± 9.9	33.2 ± 12.6
LV-EF (%)	60.1 ± 3.8	58.5 ± 5.7	53.6 ± 4.8*†	55.9 ± 3.3	57.4 ± 5.2	61.7 ± 7.4‡#
LV mass (g)	156.2 ± 33.0	163.4 ± 31.5	173.2 ± 27.3	179.8 ± 29.3	165.3 ± 31.5	154.9 ± 30.6
LV basal rotation (°)	–1.06 ± 0.67	–3.63 ± 1.13*	–8.29 ± 1.33*	–3.72 ± 2.99	–4.29 ± 2.15	–3.69 ± 2.10
LV apical rotation (°)	9.53 ± 4.84	9.53 ± 3.98	8.52 ± 3.61	3.05 ± 1.49	9.15 ± 1.87‡	15.64 ± 2.07‡#
LV twist (°)	10.60 ± 5.38	13.16 ± 3.91	16.81 ± 3.88*†	6.77 ± 3.34	13.44 ± 2.44‡	19.32 ± 2.66‡#
time-to-LV twist (ms)	268 ± 86	368 ± 139*	349 ± 58*	332 ± 148	360 ± 126	348 ± 112
${\mathrm{RA-V}}_{\text{max}}$ (mL)	50.6 ± 11.4	48.9 ± 14.4	56.4 ± 16.3	44.8 ± 10.9	50.2 ± 14.1	55.5 ± 17.9
${\mathrm{RA-V}}_{\text{preA}}$ (mL)	32.7 ± 8.3	34.0 ± 9.5	41.5 ± 10.5†	32.4 ± 8.6	35.1 ± 9.9	38.1 ± 10.8
${\mathrm{RA-V}}_{\text{min}}$ (mL)	25.1 ± 8.1	26.8 ± 9.4	31.1 ± 8.8	25.1 ± 9.3	26.9 ± 9.2	30.9 ± 8.9
TAPSE (mm)	23.0 ± 1.7	23.9 ± 3.3	23.4 ± 2.2	23.3 ± 3.3	23.8 ± 3.0	23.6 ± 2.4

*p < 0.05 vs. basal ${\mathrm{LV}}_{\text{rot}}$ < –1.79°; †*p* < 0.05 vs. –1.79° ≤ basal LV${\mathrm{LV}}_{\text{rot}}$ ≤ –6.41°; ‡*p* < 0.05 vs. apical ${\mathrm{LV}}_{\text{rot}}$ < ${\mathrm{5.33}}^{\circ }$; #*p* < 0.05 vs. ${\mathrm{5.33}}^{\circ }$≤ apical ${\mathrm{LV}}_{\text{rot}}$ ≤ ${\mathrm{13.41}}^{\circ }$. Abbreviations: LV, left ventricular; EDV, end-diastolic volume; ESV, end-systolic volume; EF, ejection fraction; RA, right atrial; $V_{\text{max}}$, end-systolic maximum RA volume; $V_{\text{preA}}$, early diastolic preatrial contraction RA volume; $V_{\text{min}}$, end-diastolic minimum RA volume; TAPSE, tricuspid annular plane systolic excursion; ${\mathrm{LV}}_{\text{rot}}$, LV rotation.

### 3.5 Degree of Apical LV Rotation and TAPSE

Similar to basal LV rotation, TAPSE did not change with the degree of apical LV rotation. While LV-EDV and basal LV rotation did not show any changes with increasing apical LV rotation, a tendentious increase of LV-EF could be detected. Similar to basal LV rotation, a tendentious increase in RA volumes could be demonstrated with increasing apical LV rotation (Table 3).

### 3.6 Correlations

No correlations were found between apical and basal LV rotations and TAPSE.

## 4. Discussion

In recent decades, echocardiography has undergone enormous technical developments, and in addition to the previously used M-mode, 2D and Doppler echocardiography, speckle-tracking (STE) and 3D echocardiography have also appeared. If the STE examination of a heart cavity takes place in a given cross-sectional plane, it is 2DSTE, if it occurs in a 3D database, it is 3DSTE [12]. Although 2DSTE is simpler to implement and does not require a special transducer, other tools and expertise, 3DSTE is closer to reality [12, 13, 14, 15, 16]. According to current professional guidelines, while LV rotational mechanics can be evaluated by 2DSTE, 3DSTE is required to accurately measure what it is validated for [12, 20, 21, 22]. Moreover, normal reference values for LV rotational parameters as assessed by 3DSTE have also been determined [17].

LV and RV not only have different embryological origins and hemodynamic environments, but their consequent morphology and contraction patterns differ as well. Moreover, RV and LV are closely related not only due to the fact that they share myocytes and ultimately the interventricular septum but due to the pericardial space itself [11]. The LV is a bullet-shaped heart cavity in the left heart, in systole its cavity becomes smaller, its walls thicken radially, shorten longitudinally and narrow circumferentially. At the same time, the LV has rotational mechanics, which is a known special form of LV movement, with the help of which it optimizes its emptying. According to physiological studies, it is responsible for 40% of the systolic ejection of the LV. Under healthy conditions, the apical region of the LV rotates counterclockwise, while the basal region of the LV rotates clockwise in systole resulting in an LV twist, which is the net sum of these movements. This occurs as a result of the simultaneous contraction of the endocardial and epicardial muscle bundles running perpendicular to each other [1, 2, 3].

The RV is the heart cavity located around the LV on the right side of the heart, it is triangular from the sides, and its cross-sectional image resembles a crescent moon, widening from the apex of the heart towards the base [4]. Although the filling and emptying of the ventricles occur at the same time and are synchronized under healthy conditions, both chambers have characteristic deformation patterns. While RV-free wall strains must be treated separately, the presence of rotational mechanics is characteristic only for LV [1, 2, 3, 11]. The muscle fibers located deep in the RV wall are responsible for the longitudinal movement from the base to the apex, as a result of which the longitudinal axis of the RV shortens and the tricuspid valve moves towards the apex. A long-known and widely accepted M-mode echocardiography-based method is the measurement of the so-called TAPSE, which characterizes this longitudinal displacement of the TA, and is related to RV contraction and shortening with a significant prognostic role [4, 5]. In addition to the above, superficially located circumferential fibers parallel to the TV are responsible for movement towards the cavity of the RV (“bellows” effect) [5, 23].

In order to better understand the coordinated operation of the ventricles, we need to know how the functions of the LV and the RV correlate with each other, e.g., the LV rotational mechanics and TAPSE, characterizing longitudinal shortening of the RV. According to the presented findings, the TAPSE value did not show any associations with basal and apical LV rotations suggesting an absence of any relationship between LV rotational mechanics and RV longitudinal shortening. Interestingly, similarly to LA, RA volumes showed a relationship with LV rotational parameters, which findings require further investigation and can be explained by strong associations between LA and RA volumetric changes [24]. However, further assessments are required to confirm the presented findings, even in certain pathologies.

### Limitations

We consider it to be important to mention the following limitations:

- Image quality is a significant limitation of 3DSTE, which is worse than that of 2DE [13, 14, 15, 16].

- Although detailed global and segmental LV strain measurements could be performed using the same 3D LV casts, our study did not aim to determine them.

- 3DSTE-derived characterization of the TA function was not aimed either.

- Moreover, volumetric and functional characterization of other cardiac chambers was not performed either.

- We did not plan to validate 3DSTE-derived LV rotational parameters [20, 21, 22] and TAPSE measurement [9] due to their validated nature.

- A one-dimensional parameter (TAPSE) does not sufficiently express the characteristics of a particular RV function. Characterizing the function of the RV with a more appropriate parameter would be much more worthy.

- Healthy volunteers were included in the study, however, neither special laboratory tests nor imaging tests were performed to rule out possible subclinical abnormalities.

## 5. Conclusions

3DSTE-derived LV rotational parameters and TAPSE are not associated suggesting that LV twist is independent of RV longitudinal shortening in healthy circumstances.

## Data Availability

The data sets generated and analyzed during the current study are not publicly available due to local restrictions but are available from the corresponding authors on reasonable request.
